# Identification of equine mares as reservoir hosts for pathogenic species of *Leptospira*

**DOI:** 10.3389/fvets.2024.1346713

**Published:** 2024-05-09

**Authors:** Camila Hamond, Emma N. Adam, Nathan E. Stone, Karen LeCount, Tammy Anderson, Ellie J. Putz, Patrick Camp, Jessica Hicks, Tod Stuber, Hans van der Linden, Darrell O. Bayles, Jason W. Sahl, Linda K. Schlater, David M. Wagner, Jarlath E. Nally

**Affiliations:** ^1^National Veterinary Services Laboratories, Animal and Plant Health Inspection Service, U.S. Department of Agriculture, Ames, IA, United States; ^2^National Centers for Animal Health Leptospira Working Group, U.S. Department of Agriculture, Ames, IA, United States; ^3^Department of Veterinary Science, University of Kentucky, Maxwell H. Gluck Equine Research Center, Lexington, KY, United States; ^4^The Pathogen and Microbiome Institute, Northern Arizona University, Flagstaff, AZ, United States; ^5^Infectious Bacterial Diseases Research Unit, Agricultural Research Service, U.S. Department of Agriculture, Ames, IA, United States; ^6^Department of Medical Microbiology and Infection Prevention, World Organisation for Animal Health (WOAH) and National Collaborating Centre for Reference and Research on Leptospirosis, Amsterdam University Medical Center, University of Amsterdam, Amsterdam, Netherlands

**Keywords:** *Leptospira*, leptospirosis, equine, Australis, *kirschneri*

## Abstract

Equine leptospirosis can result in abortion, stillbirth, neonatal death, placentitis, and uveitis. Horses can also act as subclinical reservoir hosts of infection, which are characterized as asymptomatic carriers that persistently excrete leptospires and transmit disease. In this study, PCR and culture were used to assess urinary shedding of pathogenic *Leptospira* from 37 asymptomatic mares. Three asymptomatic mares, designated as H2, H8, and H9, were PCR-positive for *lipL32*, a gene specific for pathogenic species of *Leptospira*. One asymptomatic mare, H9, was culture-positive, and the recovered isolate was classified as *L. kirschneri* serogroup Australis serovar Rushan. DNA capture and enrichment of *Leptospira* genomic DNA from PCR-positive, culture-negative samples determined that asymptomatic mare H8 was also shedding *L. kirschneri* serogroup Australis, whereas asymptomatic mare H2 was shedding *L. interrogans* serogroup Icterohaemorrhagiae. Sera from all asymptomatic mares were tested by the microscopic agglutination test (MAT) and 35 of 37 (94.6%) were seropositive with titers ranging from 1:100 to 1:3200. In contrast to asymptomatic mares, mare H44 presented with acute spontaneous abortion and a serum MAT titer of 1:102,400 to *L. interrogans* serogroup Pomona serovar Pomona. Comparison of *L. kirschneri* serogroup Australis strain H9 with that of *L. interrogans* serogroup Pomona strain H44 in the hamster model of leptospirosis corroborated differences in virulence of strains. Since lipopolysaccharide (LPS) is a protective antigen in bacterin vaccines, the LPS of strain H9 (associated with subclinical carriage) was compared with strain H44 (associated with spontaneous abortion). This revealed different LPS profiles and immunoreactivity with reference antisera. It is essential to know what species and serovars of *Leptospira* are circulating in equine populations to design efficacious vaccines and diagnostic tests. Our results demonstrate that horses in the US can act as reservoir hosts of leptospirosis and shed diverse pathogenic *Leptospira* species via urine. This report also details the detection of *L. kirschneri* serogroup Australis serovar Rushan, a species and serotype of *Leptospira*, not previously reported in the US.

## 1 Introduction

Leptospirosis is a bacterial, zoonotic, and much neglected disease that causes significant morbidity and mortality in domestic animals. The causative agents, pathogenic species of the genus *Leptospira*, are excreted via urine or found in the genital tract of domestic livestock and can survive in suitable moist environmental conditions to facilitate additional disease transmission (1, 2). To date, 41 pathogenic species of *Leptospira* have been described comprising hundreds of serovars (2–6). Human leptospirosis is estimated to cause 1.03 million cases and 58,900 deaths each year (7).

Equine leptospirosis can result in abortion, stillbirth, neonatal death, placentitis, and uveitis (1, 8, 9). Diagnostic procedures for leptospirosis fall into two groups: (1) antibody detection and (2) direct demonstration of the presence of leptospires. The microscopic agglutination test (MAT) is the antibody diagnostic assay of choice. A rising antibody titer in paired acute and convalescent sera concurrent with clinical signs of acute disease is diagnostic (1). The presence of antibody in fetal serum is diagnostic of fetal infection. Direct detection of leptospires is facilitated by molecular assays and/or culture. Culture is definitive and provides an isolate that can be comprehensively characterized by genome sequencing and serotyping. *Leptospira interrogans* serogroup Pomona serovar Pomona type kennewicki was cultured from tissues and placenta associated with aborted equine fetuses in the US (10–14).

In Europe, serogroup Pomona has been identified as a cause of equine abortion as have the serogroups Australis, Hebdomadis, and Icterohaemorrhagiae (15). In Europe, equine recurrent uveitis is associated with serogroups Grippotyphosa, Australis, Sejroe, Pomona, and Javanica (16, 17). Horses in Europe have also been identified as reservoir hosts of *Leptospira*, asymptomatic carriers that persistently shed live pathogenic *Leptospira* via urine into the environment to maintain disease transmission (18). In Northern Ireland, it is hypothesized that horses act as a reservoir host for *L. interrogans* serogroup Australis serovar Bratislava, which has been cultured from equine kidneys (19). Seroprevalence studies on horses throughout the world demonstrate high levels of reactivity with serogroup Australis serovar Bratislava, which is used to further support the role of horses serving as reservoir hosts for *L. interrogans* serogroup Australis serovar Bratislava on a global level (20–25).

The MAT serological assay cannot diagnose horses acting as reservoir hosts of infection since seroprevalence studies in normal equine populations demonstrate exposure, not active clinical infection (1, 21, 26, 27). Direct detection of leptospires by culture or molecular methods is required to identify asymptomatic reservoir hosts of infection (1, 28, 29). However, culture is not routinely performed due to the fastidious growth requirements of *Leptospira*, the length of time, and the high levels of expertise required (30, 31). To design efficacious vaccination and diagnostic strategies for equine leptospirosis, it is essential to identify infected animals and determine both the genotype (species) and phenotype (serogroup/serovar) of *Leptospira* species associated with equine infections. Successful culture provides isolates that can be comprehensively characterized and used in bacterin vaccines. Since there are no published reports confirming that horses in the US act as reservoir hosts of leptospirosis, a population of asymptomatic thoroughbred mares was screened by molecular assays to determine if any were shedding *Leptospira* via urine. Samples from positive mares were then further examined by culture and *Leptospira* genome enrichment techniques to identify the species and serotype of *Leptospira* associated with subclinical carriage and for comparison with a strain of *Leptospira* commonly associated with overt clinical infection in horses.

## 2 Materials and methods

### 2.1 Samples

A total of 37 asymptomatic thoroughbred mares from farm #1 in central Kentucky with no history of leptospirosis were sampled from December 2022 to February 2023. Blood was collected by jugular venipuncture into serum separator tubes (Vacutainer^®^, BD Diagnostics, Franklin Lakes, NJ, United States). Urine for PCR was collected by free catch and chilled on ice. In brief, the tail was wrapped, and sterile water used to remove all visible debris and thoroughly cleaned the vulva and surrounding area. Mid-stream urine was collected into sterile containers and shipped on ice packs for processing by PCR within 24 h. To collect urine for culture, if a mare was PCR-positive, the tail was wrapped and pulled away from the perineal area. Sterile water was used to remove all visible debris and thoroughly clean the vulva and surrounding area. The vulvar region was cleaned lightly with 70% ethyl alcohol and gently patted dry. The process was repeated if any contamination of the site occurred prior to urination. A diuretic was administered by intravenous injection (furosemide 5% injectable, 1 mg/kg based on estimated weight of mare), and midstream urine from successive voids was collected into sterile containers. The urine was passed to an assistant with clean gloves, and a sterile pipette was used to transfer 1 mL of urine from each voided sample to separate conical tubes containing 9 mL of HAN medium containing 5-fluorouracil (100 μg/μL) (30). Remaining urine from this second collection was transported on ice packs for a repeat PCR within 24 h.

Samples from farm #2 in central Kentucky, a farm with a very recent history of abortion by *Leptospira* as diagnosed by the University of Kentucky Veterinary Diagnostic Laboratory, were collected from a single mare suffering from an abortion in the final trimester of gestation (16 January 2023). Urine, chorioallantoic membrane, amnion, and allantoic fluid were immediately shipped on ice packs for processing within 24 h. A second set of samples collected 2 days later (18 January 2023), including urine, exudate, and a uterine swab, were inoculated directly into HAN medium and immediately shipped for processing within 24 h.

### 2.2 Microscopic agglutination test

The MAT was performed using a panel of 18 antigens representative of 15 serogroups (Supplementary Table S1), as previously described (32, 33). A titer was considered positive at ≥1:100. Sera were also tested for reactivity in the MAT with the strain H9, an isolate recovered from a mare in this study.

### 2.3 Molecular detection of pathogenic *Leptospira*

The gene encoding the major outer membrane protein LipL32 discriminates pathogenic species of *Leptospira* from saprophytes. Before testing equine urine samples by *lipL32* rtPCR (34, 35), preliminary studies were performed with spiked equine urine to optimize parameters for equine urine storage and pre-processing. In brief, 1 mL of *L. borgpetersenii* serovar Tarassovi strain Perepelitsin, at a density of 10^8^ leptospires/mL, was inoculated into 9 mL of freshly collected equine urine. A 1 mL aliquot of this was serially diluted 10-fold to prepare spiked samples containing 10^7^ *Leptospira*/mL to 10^0^ *Leptospira*/mL urine. Intact motile leptospires were enumerated by dark-field microscopy as previously described (36). In total, 1 mL of each dilution was then processed for the extraction of DNA after storage in various conditions/time points, including: (A) that same day, (B) that same day after centrifugation at 900 × g for 10 min to remove “sludge,” (C) after storage for 24 h in a Styrofoam container with ice packs, (D) after storage for 24 h in a Styrofoam container with ice packs followed by centrifugation to remove “sludge,” (E) after storage for 48 h in a Styrofoam container with ice packs, (F) after storage for 48 h in a Styrofoam container with ice packs followed by centrifugation to remove “sludge,” (G) after freezing at −20°C for 24 h, and (H) after samples had been centrifuged at 900 × g to remove “sludge” and stored at −20°C for 24 h. Storage treatments A to H correlate with labels are shown in Supplementary Figure S1. Thereafter, urine was centrifuged at 12,000 × g for 30 min. The supernatant was removed, and the pellets were washed twice by resuspending in 1 mL phosphate buffered saline (PBS) and centrifuging at 12,000 × g for 15 min, leaving the final pellets in ~100 μL. DNA was extracted from the urinary pellet using the Maxwell RSC Purefood Purification Pathogen Kit (Promega Corporation, Madison, Wisconsin, United States), following the manufacturer’s instructions, except using a 1 h incubation with 200 μL lysis buffer A and a 100 μL elution volume (37). *lipL32* rtPCR was performed using 10 μL of Perfect taq qPCR ToughMix low ROX^™^ (Quantabio, Beverly, MA, United States), 400 nmol/L of each primer, 132.5 nmol/L of probe, TaqMan^™^ Exogenous Internal Positive Control Reagents: 2 μL of 10X Exo IPC Mix, 0.4 μL of 50X Exo IPC DNA, and 5 μL of DNA extract from culture or sample. rtPCR cycling was conducted on a QuantStudio^™^ 7 (Thermo Fisher Scientific, United States) starting with an initial 3 min denaturation at 95°C for Taq polymerase activation, followed by 40 cycles of denaturation at 95°C for 15 s and primer annealing and extension at 60°C for 1 min. PCR of samples was performed in triplicate and considered positive by *lipL32* rtPCR when duplicate or triplicates were positive with Ct values <40, as previously described (34, 35). DNA from *L. borgpetersenii* serovar Tarassovi strain Perepelitsin was used to prepare a standard curve as previously described (38).

A 45 mL aliquot of urine from each asymptomatic mare was centrifuged at 900 × g for 10 min at 4°C, and the supernatant was transferred to a clean 50 mL conical tube. Urine was then centrifuged at 12,000 × g for 30 min at 4°C. The supernatant was removed, and the pellet washed twice by resuspending in 1 mL of PBS and centrifuging at 12,000 × g for 10 min at 4°C. DNA was extracted as described above, and *lipL32* rtPCR was performed as previously described (34, 35).

### 2.4 Culture

A 1 mL aliquot of freshly collected voided urine was immediately inoculated into 9 mL of HAN medium (30) and transported to the National Animal Disease Center (NADC), Ames, for culture of *Leptospira*, as previously described (37). Two 10-fold serial dilutions were made from the initial inoculum (i.e., 500 μL) into 5 mL of liquid HAN medium and incubated at 37°C in 5% CO_2_. Vulvar exudate and chorioallantois samples were vortexed with 9 mL of HAN medium and two 10-fold serial dilutions were made (i.e., 500 μL) into 5 mL of liquid HAN at 37°C in 5% CO_2_. Inoculated tubes were examined daily by darkfield microscopy for the first 2 weeks and then periodically for 6 months.

### 2.5 Molecular typing of *Leptospira* isolates

DNA was extracted from a 5 mL culture of the isolated strains using the Maxwell RSC Purefood Purification Pathogen Kit (Promega Corporation, Madison, WI), following the manufacturer’s instructions. The genomic DNA concentration was determined by Qubit (Qubit dsDNA Broad Range Assay Kit, Qubit 3.0 fluorometer, Invitrogen, Carlsbad, CA, United States). Illumina whole-genome sequence (WGS) was obtained (Nextera XT DNA Library Preparation Kit and the MiSeq Sequencer, 2 × 250 v2 paired-end chemistry, Illumina, San Diego, CA, United States), according to the manufacturer’s instructions. Illumina WGS reads were taxonomically identified using Kraken 2 version 2.1 (39). Reads were assembled with SPAdes 3.13 (40) and verified by comparing the expected genome size with the actual assembly size and verifying contigs as *Leptospira* by BLASTN (41) against the NCBI nucleotide (NT) database.

### 2.6 Serotyping of *Leptospira* isolates

The serogroup of strains H9 and H44 were determined by the MAT method using a panel of polyclonal rabbit reference antisera representing 13 serogroups (Supplementary Table S2). The serovar of strains H9 and H44 was determined by performing MAT with panels of monoclonal antibodies (mAbs) that characteristically agglutinate serovars from the serogroups Australis and Pomona, respectively, as previously described (42).

### 2.7 DNA capture and enrichment

DNA from two *Leptospira* PCR-positive culture-negative urine samples (designated as H2 and H8) was subjected to pan-pathogenic *Leptospira* DNA capture and enrichment, as previously described (43). The two samples were processed differently because DNA from sample H8 was highly fragmented and displayed low concentrations of nucleic acids (~0.5 ng/μL), whereas sample H2 had DNA that was more intact (average fragment size >6,000 bp) and concentrated (~4 ng/μL). For sample H2, the DNA was diluted to ~2 ng/μL in a volume of 40 μL and sonicated to an average size of 228 bp using a Q800R2 sonicator (QSonica, Newtown, CT, United States). For sample H8, 40 μL of undiluted DNA was subjected to brief sonication, and the final average fragment size was 103 bp. Short-read next-generation libraries were prepared separately using Agilent Sure-Select methodology. The libraries were then pooled together in equimolar amounts and were subjected to one round of DNA capture and enrichment and then sequenced on an Illumina MiSeq instrument using a MiSeq v3 600 cycle kit (2 × 300bp reads).

To estimate the percentage of *Leptospira* reads in the enriched sequences, reads were mapped against the standard Kraken database with Kraken v2.1.2 (39). Reads assigned as *Leptospira* were then extracted and assembled using SPAdes v3.13.0 (40) with default settings; assemblies were also generated for reads that were generated from isolates for samples H9 and H44. Assemblies for H2, H8, H9, and H44 were placed into a genus dendrogram containing 66 *Leptospira* reference genomes with Mashtree v1.2.046 (44) to confirm species identification. GenBank accession numbers for each genome are presented in figures.

### 2.8 Read mapping and phylogenomics

Single nucleotide polymorphisms (SNPs) were identified among two enriched genomes (H2 and H8), two genomes from cultured isolates (H9 and H44), and 103 publicly available *Leptospira* genomes (GenBank accession numbers for each genome are presented in figures) by aligning reads against reference genomes *Leptospira interrogans* serogroup Canicola serovar Canicola strain LJ178 (GCA_008831445.1), *Leptospira interrogans* serogroup Icterohaemorrhagiae serovar Copenhageni strain Fiocruz_L1-130 (GCA_000007685.1), or *Leptospira kirschneri* serogroup Grippotyphosa serovar Grippotyphosa strain RedPanda1 (GCA_027563495.1) using minimap2 v2.22 (45) and calling SNPs from the BAM file with GATK v4.2.2 (46) using a depth of coverage ≥3× and a read proportion of 0.9. Based on a reference self-alignment with NUCmer v3.1 (47), SNPs that fell within duplicated regions were filtered from downstream analyses. All of these methods were wrapped by NASP v1.2.1 (48). Maximum likelihood phylogenies were then inferred on the concatenated SNP alignments using IQ-TREE v2.2.0.3 with default parameters (49), 1,000 bootstrap replicates, and the integrated ModelFinder method (50); the phylogenies were rooted with either *Leptospira interrogans* serovar Canicola strain LJ178, *Leptospira interrogans* serovar Copenhageni strain Fiocruz_L1-130, or *Leptospira kirschneri* serovar Grippotyphosa strain RedPanda1, as appropriate. To determine breadth of coverage for the enriched genomes, reads were aligned against *Leptospira interrogans* serovar Copenhageni strain Fiocruz_L1-130 for sample H2 and *Leptospira kirschneri* serovar Grippotyphosa strain RedPanda1 for sample H8 with minimap2, and the per base depth of coverage was calculated with Samtools v1.6 (51).

### 2.9 Evaluation of virulence

All animal experimentation was conducted in accordance with protocols as reviewed and approved by the Animal Care and Use Committee at the NADC and USDA institutional guidelines. Strains H9 and H44 were propagated in liquid HAN medium at 29°C and were evaluated for virulence by intraperitoneal injection of 10^8^ leptospires in 500 μL into groups (*n* = 4 per group) of golden Syrian hamsters (*Mesocricetus auratus*). A negative control group (*n* = 4) received HAN medium alone. After 3 weeks of inoculation, animals were euthanized and exsanguinated by cardiac puncture, and whole blood smears were evaluated. Dried slides were submitted to the National Animal Disease Center Microscopy Services laboratory for Giemsa staining as reported previously (52), and Giemsa-stained slides were evaluated by counting the first 100 white blood cells to identify the portion of foamy macrophages. Differential cell counts were evaluated in R (53), where a linear regression model fitting challenge was used as a fixed effect to generate least square means and standard errors. Kidney and liver tissues were harvested for culture. qPCR for *lipL32* was performed on liver and kidney samples, as previously described (54), except using DNA from *L. interrogans* serovar Canicola strain Hond Utrecht IV for a standard curve. Sera were collected for MAT, which was performed according to World Organisation for Animal Health (WOAH) guidelines (32), using a panel of 18 antigens representative of 15 serogroups (Supplementary Table S1), as well as strains H9 and H44. A titer was considered positive at ≥ 1:100.

### 2.10 Electrophoresis and immunoblots

In addition to strains H9 and H44, *L. interrogans* serogroup Icterohemorrhagiae serovar Copenhageni strain Fiocruz_L1-130 (55, 56) and *L. interrogans* serogroup Australis serovar Bratislava strain PigK151 (57) were propagated in HAN medium until mid-late log phase and harvested by centrifugation (10,000 × g, 4°C, 30 min), washed twice with PBS, and processed for one-dimensional (1-D) sodium dodecyl-sulfate polyacrylamide gel electrophoresis (SDS-PAGE) on 12% acrylamide gels (Bio-Rad, Hercules, CA, United States), according to the manufacturer’s guidelines. Proteins were visualized by staining with SYPRO Ruby (Invitrogen), and LPS was visualized by staining with Pro-Q Emerald 300 (Invitrogen), according to the manufacturer’s guidelines. For immunoblotting, samples were transferred by semi-dry transfer (Amersham TE77 PWR) to an Immobilon-P transfer membrane (Millipore, 220 Bedford, MA, United States) and blocked overnight at 4°C with Starting Block (PBS) blocking buffer (Thermo Fisher). Membranes were individually incubated with indicated antisera [anti-LipL32, anti-Loa22 or anti-LipL21 (58, 59)] or reference antisera [anti-Pomona, anti-Bratislava, anti-Copenhageni, or anti-Ramisi (NVSL, USDA, Ames, IA)] diluted in blocking buffer followed by incubation with horseradish-peroxidase anti-rabbit immunoglobulin G (Sigma, St. Louis, MO, United States) conjugate diluted 1:4,000 in blocking buffer. Bound conjugates were detected using Clarity Western ECL substrate (Bio-Rad), and images were acquired using a Bio-Rad ChemiDoc MP imaging system.

## 3 Results

### 3.1 Detection of pathogenic *Leptospira*

PCR of equine urine samples that were centrifuged at 900 × g for 10 min to remove “sludge” and processed on the same day or within 24 h of storage at 4°C provided the lowest limits of detection at 10 *Leptospira*/mL of urine (Supplementary Figure S1). Storage of samples at 4°C for 48 h, freezing, or not removing “sludge” by centrifugation at 900 × g for 10 min increased the limit of detection to 10^2^ *Leptospira*/mL of urine. Freezing samples without removing “sludge” increased the limit of detection to 10^3^ *Leptospira*/mL of urine.

Urine from 37 asymptomatic mares, designated as H1 to H37, was tested by rtPCR for *lipL32* using optimized protocols as described above. Three urine samples (8%), designated as H2, H8, and H9 from three different pregnant mares, were PCR-positive with Ct values of 37, 38.2, and 36.9, respectively (Table 1). Repeat testing of urine collected 4 days later was PCR-positive for mares H8 and H9 but negative for mare H2. Urine from mare H9 was culture-positive for *Leptospira*. Complete data on all equine samples are shown in Supplementary Table S3.

**Table 1:** Detection of *Leptospira* in urine from asymptomatic mares by real-time PCR and culture.

Horse number	12/1/2023	12/5/2023		
	rtPCR (Ct)	rtPCR Urine void 1 (Ct)	rtPCR Urine void > 1 (Ct)	Culture
H2	37*	Negative	Negative	Negative
H8	38.2	36.9	35*	Negative
H9	36.9	Negative	38	Positive

Date of sample collection is provided, as Ct values are for PCR-positive urine samples. *Indicates PCR-positive urine samples used for DNA enrichment.

Urine, chorioallantoic membrane, amnion, and allantoic fluid collected from a mare (designated as H44) showing clinical disease, and in the process of aborting at the time of sample collection, were positive by rtPCR for *lipL32* with Ct values of 26, 31.8, 29.9, and 25.4, respectively (Supplementary Table S3). Chorioallantoic membrane was culture-positive for *Leptospira*. Samples collected from mare H44 after 2 days post-abortion, including urine, vulvar exudate, and uterine swabs, were all positive by rtPCR for *lipL32*, but all cultures were heavily contaminated (Supplementary Table S3).

### 3.2 Genotyping and serotyping of isolates of *Leptospira*

**Figure 1:**
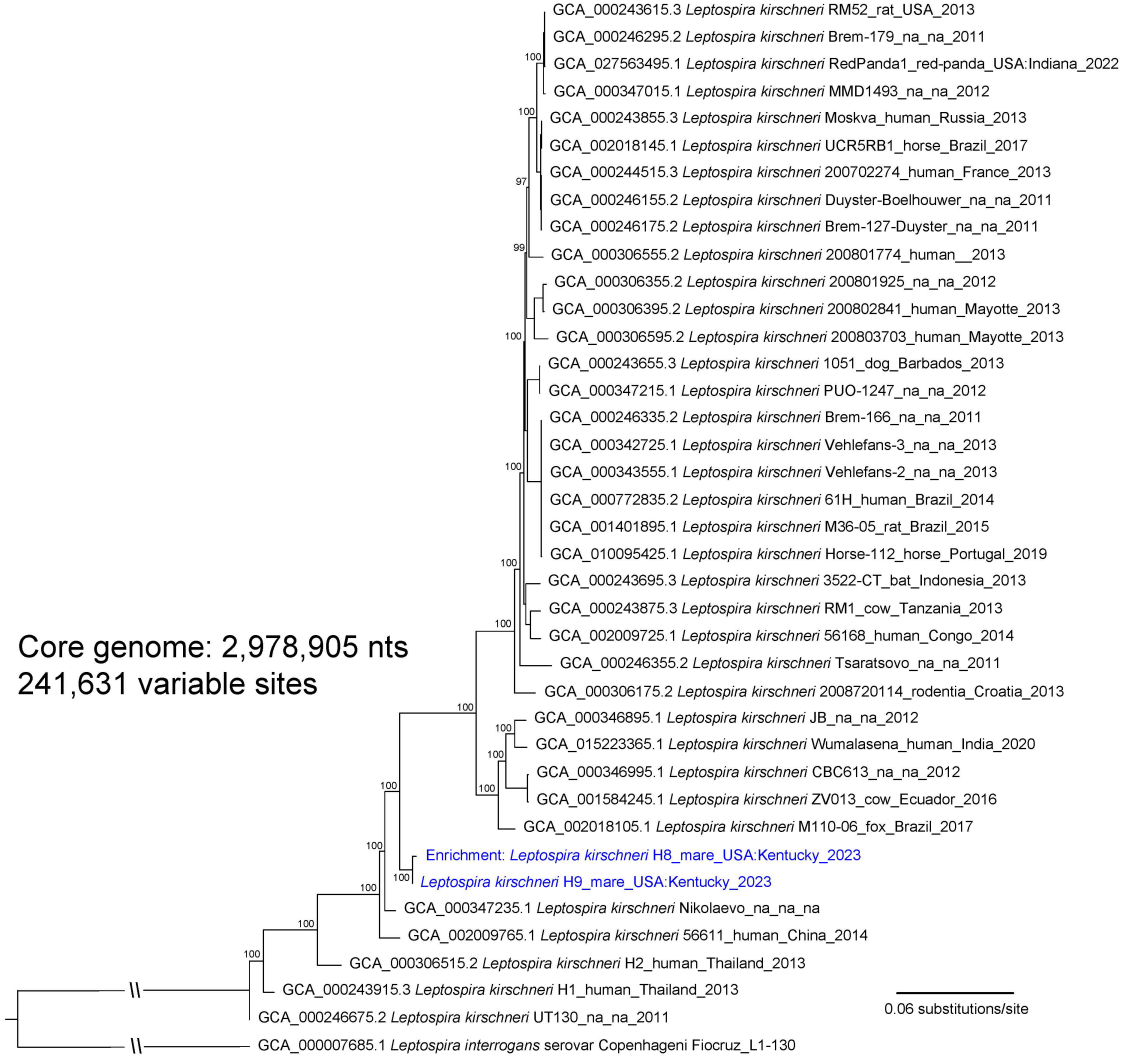
A maximum likelihood phylogeny of 36 *L. kirschneri* reference genomes together with *Leptospira* genomic DNA obtained from mares H8 and H9 (highlighted with blue text). The tree was inferred by IQ-TREE from a concatenated SNP alignment of 241,631 positions out of a core genome size of 2,978,905 nts. *Leptospira* from these two animals cluster together in a distinct *L. kirschneri* clade. GenBank accession numbers for reference genomes are included in the annotations along with strain name, host, geographic location, and year of collection when available. The phylogeny is rooted with *L. interrogans* strain Fiocruz_L1-130, and bootstrap values based on 1,000 replicates are indicated at major nodes.

An isolate of *Leptospira* cultured from urine of asymptomatic mare H9, designated as strain H9, was genotyped as *L. kirschneri* (Figure 1; Supplementary Figure S2). Serotyping of strain H9 with reference antisera identified the isolate as belonging to serogroup Australis (Supplementary Table S3). Additional serotyping with monoclonal antibodies to identify serovar confirmed that strain H9 was most similar to serovar Rushan due to similar reactivity patterns with the Rushan reference strain within the serogroup Australis (Supplementary Figure S3). Strain H9 is classified as *L. kirschneri* serogroup Australis serovar Rushan.

**Figure 2:**
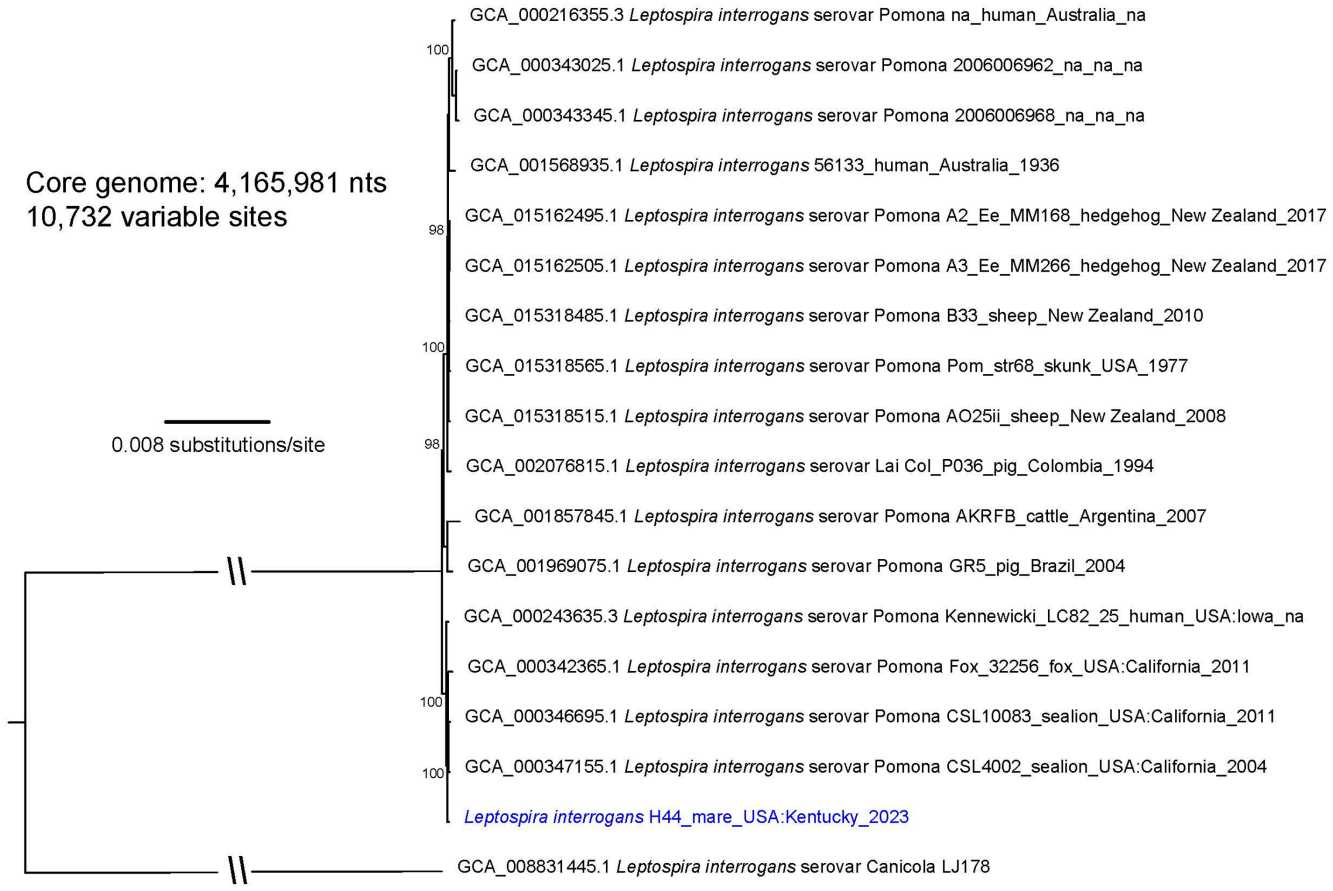
A maximum likelihood phylogeny of 16 *L. interrogans* reference genomes that fall within the “serovar Pomona” clade together with *Leptospira* strain H44 (highlighted with blue text). The tree was inferred by IQ-TREE from a concatenated SNP alignment of 10,732 positions out of a core genome size of 4,165,981 nts. *Leptospira* from this animal cluster among four other serovar Pomona genomes obtained from two sea lions, a fox, and a human from the US GenBank accession numbers for reference genomes are included in the annotations along with strain name, host, geographic location, and year of collection when available. The phylogeny is rooted with *L. interrogans* serovar Canicola strain LJ178, and bootstrap values based on 1,000 replicates are indicated at major nodes.

An isolate of *Leptospira* cultured from chorioallantois of mare H44 presenting with abortion was genotyped as *L. interrogans* (Figure 2; Supplementary Figure S2). Serotyping of strain H44 with reference antisera identified the isolate as belonging to serogroup Pomona (Supplementary Table S3). Additional serotyping with monoclonal antibodies to identify serovar confirmed that strain H44 was most similar to serovar Pomona due to similar reactivity patterns with the Pomona reference strain within the serogroup Pomona (Supplementary Figure S4). Strain H44 is classified as *L. interrogans* serogroup Pomona serovar Pomona.

### 3.3 DNA capture and enrichment

Given the fastidious growth requirements of pathogenic *Leptospira* and the inherent difficulties with their culture from urine of large animal species, urine samples that were PCR-positive for *lipL32* but culture-negative for *Leptospira* were processed using a culture-independent DNA capture and enrichment system to obtain *Leptospira* genomic information directly from urine samples H2 and H8. Enriched samples H2 and H8 had very high and moderate proportions, respectively, of sequencing reads that assigned to *Leptospira*: 98.36% for H2 (1,529,676 of 1,555,190 reads) and 60.08% for H8 (562,885 of 936,958 total reads). Sample H2 revealed a breadth of coverage of 99.95% with an average depth of 77.5× (72–78×) against reference genome *L. interrogans* serogroup Icterohemorrhagiae serovar Copenhageni strain Fiocruz_L1-130. Sample H8 displayed a breadth and depth of coverage of 92.72% and 32.5× (3–176×) when aligned against *Leptospira kirschneri* serogroup Grippotyphosa serovar Grippotyphosa strain RedPanda1. The whole genome *Leptospira* dendrogram placed enriched and isolate genomes for strains H8 and H9 in the *L. kirschneri* clade, whereas enriched and isolate genomes for strains H2 and H44 grouped with *L. interrogans* (Supplementary Figure S2). To obtain higher resolution within *L. interrogans*, a core genome phylogeny was constructed containing enriched genome H2 and isolate genome H44 plus 51 diverse *L. interrogans* genomes (Supplementary Figure S5). This analysis placed H44 within the “serovar Pomona” clade and H2 within the “serovar Copenhageni” clade.

**Figure 3:**
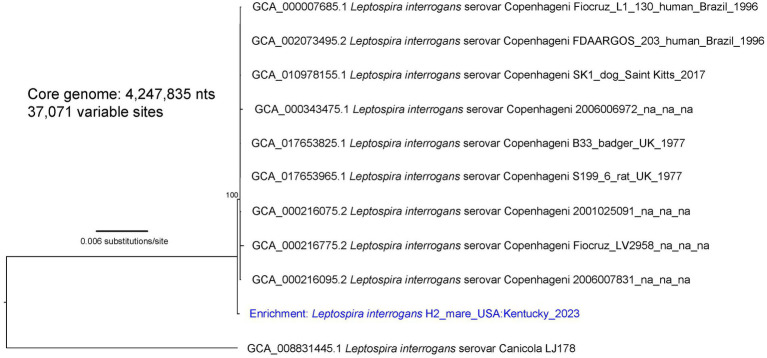
A maximum likelihood phylogeny of nine *L. interrogans* reference genomes that fall within the “serovar Copenhageni” clade together with enriched *Leptospira* genomic DNA from mare H2 (highlighted with blue text). The tree was inferred by IQ-TREE from a concatenated SNP alignment of 37,071 positions out of a core genome size of 4,247,835 nts. *Leptospira* from this animal is mostly similar to other genomes from the “serovar Copenhageni” clade, yet it is distinct. GenBank accession numbers for reference genomes are included in the annotations along with strain name, host, geographic location, and year of collection when available. The phylogeny is rooted with *L. interrogans* serovar Canicola strain LJ178, and bootstrap values based on 1,000 replicates are indicated at major nodes.

Three separate phylogenies were constructed based on the core genomes of: (1) enriched genome H8, isolate genome H9, and 36 *L. kirschneri* reference genomes (Figure 1); (2) isolate genome H44 and 16 *L. interrogans* reference genomes that assign to the “serovar Pomona” clade (Figure 2); and (3) enriched genome H2 and 9 *L. interrogans* reference genomes that fall within the “serovar Copenhageni” clade (Figure 3). Genomes for strains H8 and H9 clustered together in a clade within, yet distinct from, other *L. kirschneri* genomes (Figure 1). Genome H44 grouped with other genomes of *L. interrogans* serovar Pomona from US isolates of *Leptospira* derived from a fox, sea lions, and a human (Figure 2). Finally, enriched genome H2 was most like other *L. interrogans* serovar Copenhageni genomes (Supplementary Figure S5), yet still distinct by displaying 376 SNP differences to separate it from the “serovar Copenhageni” clade (Figure 3).

### 3.4 Microscopic agglutination test

Sera from 37 asymptomatic mares were tested by MAT. Of these, 35 (94.6%) were seropositive (titer ≥1:100). Equivalent highest titers were observed for more than one serogroup in 10 samples (Supplementary Table S3). The most frequent highest-reacting MAT titer was with serogroup Australis (31.4%), followed by serogroups Pomona (20%), Djasiman (5.7%), Grippotyphosa (5.7%), and Icterohaemorrhagiae (2.8%). All equine sera were seronegative when tested by the MAT using strain H9.

Serum from the clinically infected mare (H44) presenting with abortion had a high MAT titer to serogroup Pomona (1:102,400).

### 3.5 Evaluation of virulence

**Figure 4:**
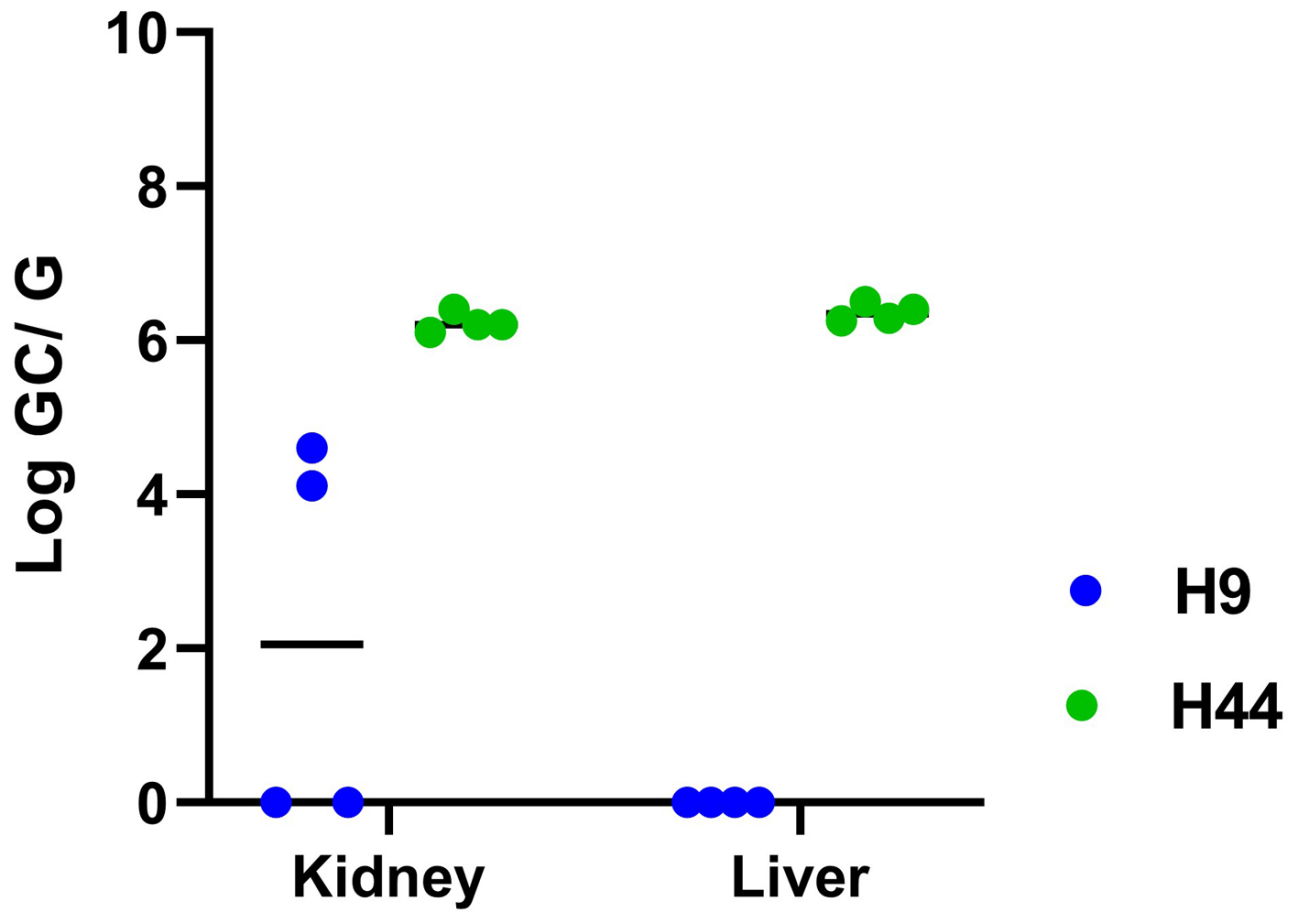
Detection of *Leptospira* in the kidney and liver of hamsters after inoculation with *L. kirschneri* serogroup Australis strain H9 (associated with asymptomatic carriage in a mare) and *L. interrogans* serogroup Pomona strain H44 (associated with spontaneous equine abortion) by qPCR detection of *lipL32*. Numbers of genome copies (GC) of leptospires per gram (G) of tissue are shown in Table 2.

Experimentally inoculated hamsters did not show any clinical signs of infection or weight loss after intraperitoneal inoculation with *L. kirschneri* serogroup Australis strain H9. Similarly, hamsters inoculated with *L. interrogans* serogroup Pomona strain H44 did not show clinical signs of disease, except for one hamster that was losing weight and euthanized at day 14 post-inoculation. After 3 weeks of infection, all remaining hamsters were euthanized. All kidney samples from each group tested positive by *lipL32* qPCR (Table 2 and Figure 4). Livers from hamsters inoculated with strain H44 were also PCR-positive but livers from hamsters inoculated with strain H9 were PCR-negative. Kidneys and livers from hamsters inoculated with strain H44 were all culture-positive, but only two of four hamsters inoculated with strain H9 were kidney-culture positive (Table 2). Hamsters inoculated with *L. kirschneri* serogroup Australis strain H9 had positive MAT titers against strain H9 and reference strain *L. interrogans* serogroup Australis serovar Bratislava strain Jez Bratislava but were seronegative when tested against other reference antigens (Table 2). Hamsters inoculated with *L. interrogans* serogroup Pomona strain H44 had positive MAT titers against strain H44 and reference strain *L. interrogans* serogroup Pomona serovar Pomona strain Pomona but were seronegative when tested against other reference antigens (Table 2). Manual blood count differentials determined that all hamsters challenged with both H9 and H44 produced circulating foamy macrophages (1.50 ± 0.66 and 1.55 ± 0.66, respectively, foamy macrophages per 100 white blood cells evaluated), a marker associated with virulence and disease severity in the hamster model of leptospirosis (52, 60).

**Table 2:** Hamster challenge with *L. kirschneri* serogroup Australis strain H9 and *L. interrogans* serogroup Pomona strain H44.

Challenge strain	Hamster number	MAT titer with Serogroup Australis		MAT titer with Serogroup Pomona		Kidney		Liver	
		Reference strain Jez Bratislava	Strain H9	Reference strain Pomona	Strain H44	Culture	qPCR (GC/g)	Culture	qPCR (GC/g)
*L. kirschneri* serogroup Australis strain H9	1	200	1,600	Negative	Negative	Negative	1.1	Negative	0
	2	Negative	800	Negative	Negative	Negative	1.35	Negative	0
	3	800	1,600	Negative	Negative	Positive	4.6	Negative	0
	4	200	3,200	Negative	Negative	Positive	4.11	Negative	0
*L. interrogans* serogroup Pomona strain H44	5	Negative	Negative	800	1,600	Positive	6.2	Positive	6.4
	6	Negative	Negative	800	1,600	Positive	6.4	Positive	6.5
	7	Negative	Negative	1,600	3,200	Positive	6.1	Positive	6.29
	8	Negative	Negative	1,600	3,200	Positive	6.22	Positive	6.25

MAT reciprocal titers are provided, as are results of culture and qPCR which lists numbers of genome copies (GC) of leptospires detected per gram (g) of tissue. All data are based on horse samples and their respective analyses.

### 3.6 Protein and lipopolysaccharide profiles of *Leptospira*

**Figure 5:**
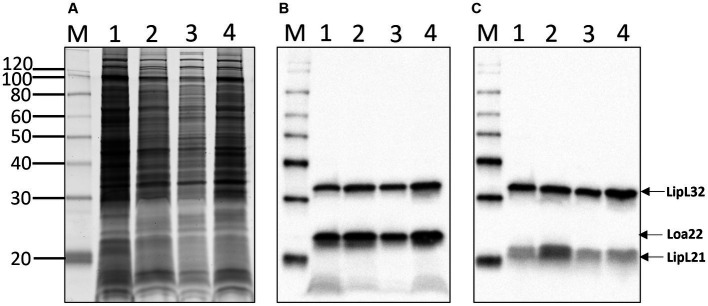
Total protein profiles **(A)** of 1: *L. kirschneri* serogroup Australis strain H9, 2: *L. interrogans* serogroup Pomona strain H44, 3: *L. interrogans* serogroup Icterohaemorrhagiae serovar Copenhageni strain Fiocruz_L1-130, and 4: *L. interrogans* serogroup Australis serovar Bratislava strain PigK151 and immunoblotting with **(B)** anti-LipL32/Loa22 or **(C)** anti-LipL32/LipL21. Molecular mass markers are indicated.

Total protein profiles of *L. kirschneri* serogroup Australis strain H9 and *L. interrogans* serogroup Pomona strain H44 were compared with those of *L. interrogans* serogroup Icterohemorrhagiae serovar Copenhageni strain Fiocruz_L1-130 and *L. interrogans* serogroup Australis serovar Bratislava strain PigK151 (Figure 5A). As expected for pathogenic *Leptospira*, similar protein profiles were detected across different species and serovars, and all strains were confirmed to express the pathogen-associated protein LipL32 and known virulence factors Loa22 and LipL21 (Figures 5B,C).

**Figure 6:**
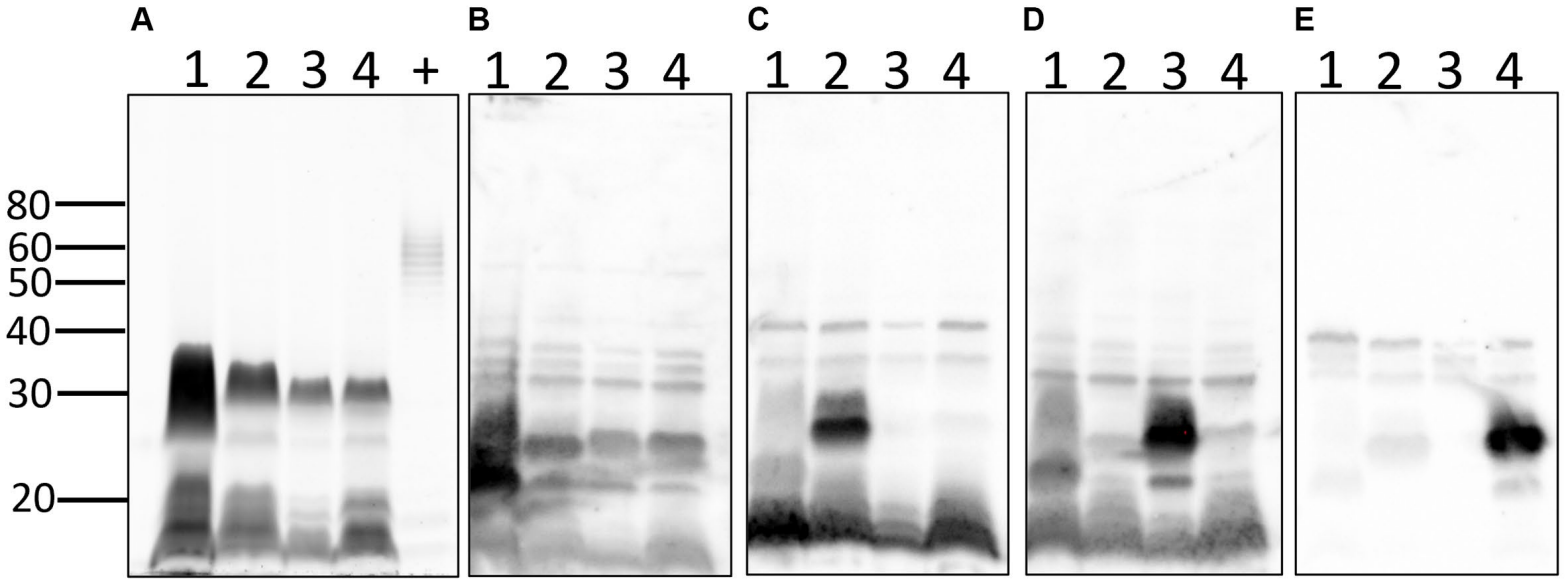
Lipopolysaccharide profiles of leptospires: total LPS profiles **(A)** of 1: *L. kirschneri* serogroup Australis strain H9, 2: *L. interrogans* serogroup Pomona strain H44, 3: *L. interrogans* serogroup Icterohaemorrhagiae serovar Copenhageni strain Fiocruz_L1-130, 4: *L. interrogans* serogroup Australis serovar Bratislava strain PigK151, and positive LPS control (+) comprising 10 μg of LPS from *E. coli* serotype 055:B5. Immunoblotting with reference antisera against **(B)** serogroup Australis serovar Ramisi, **(C)** serogroup Pomona serovar Pomona, **(D)** serogroup Icterohaemorrhagiae serovar Copenhageni, and **(E)** serogroup Australis serovar Bratislava. Molecular mass markers are indicated.

Total lipopolysaccharide (LPS) profiles of each strain were also compared (Figure 6A). The results confirm the unusual and atypical LPS profile of pathogenic *Leptospira* compared with that of *E. coli* and confirm different LPS profiles between serovars of *Leptospira* from different serogroups. Antigenic differences of LPS between serogroups and different serovars within the same serogroup were confirmed by immunoblotting with reference antisera specific for (1) serogroup Australis serovar Ramisi, (2) serogroup Pomona serovar Pomona, (3) serogroup Icterohemorrhagiae serovar Copenhageni, and (4) serogroup Australis serovar Bratislava (Figures 6B–E).

### 3.7 Data availability

Sequencing data and accession numbers are available at NCBI under BioProject ID PRJNA994138.

## 4 Discussion

The primary goal of this study was to determine whether asymptomatic mares can act as reservoir hosts of infection, and if so, what species and serovars of *Leptospira* are involved. Our approach was limited to the goodwill and cooperation of management and staff of a single commercial thoroughbred breeding farm that facilitated collection of samples from a limited number of available mares, with an incomplete vaccination history, on a single farm, within a relatively short timeframe. Our results demonstrate that asymptomatic mares can shed different species and serovars of *Leptospira* via urine. Unexpectedly, a single isolate recovered from an asymptomatic mare is classified as belonging to *L. kirschneri* serogroup Australis serovar Rushan, which has not previously been reported in the US or in horses. These findings justify more comprehensive studies on horses throughout the US to accurately determine the prevalence of equine leptospirosis, and species and serovars of *Leptospira* are associated with asymptomatic shedding.

The literature is replete with serological evidence that horses are exposed to serovars within the serogroup Australis and coupled with the isolation of *L. interrogans* serogroup Australis serovar Bratislava from horses in Ireland and Portugal (18, 19); it is hypothesized that horses act as reservoir hosts for serovar Bratislava. However, serology by MAT alone does not detect or reflect shedding of *Leptospira* in reservoir hosts of leptospirosis (1). Therefore, urine samples from asymptomatic mares were initially screened by rtPCR targeting *lipL32*, a gene present in pathogenic *Leptospira* species that discriminates from saprophytic species. Optimal detection of *lipL32* in equine urine requires that they are processed for PCR within 24 h and shipped on icepacks (Supplementary Figure S1). Three PCR-positive mares (designated as H2, H8, and H9) were identified and targeted for culture and repeat rtPCR, of which two (H8 and H9) remained PCR-positive and one (H9) was culture-positive, validating initial results. Intermittent shedding of leptospires in urine of reservoir hosts is not uncommon, which may account for the negative rtPCR in mare H2 (38, 61). Because the farm’s veterinary clinician initiated antimicrobial therapy after PCR-positive results became available, additional rtPCR and culture were not performed. Culture of *Leptospira* from urine of large domestic animals is notoriously difficult (1), so a culture-independent approach for characterizing the genome of *Leptospira* in PCR-positive samples was used to genomically characterize those species associated with subclinical infection in mares H2 and H8 (43). Mare H2 was determined to be shedding *L. interrogans*, the genome of which aligned most closely with serogroup Icterohemorrhagiae serovar Copenhageni but still quite distinct, with 376 SNPs separating it from other genomes in this clade (Figure 3). Serogroup Icterohemorrhagiae is a leading cause of acute leptospirosis in human patients, and its classic reservoir host is the rat, not domestic animals. However, this serogroup has been isolated from equine kidney and aborted fetuses previously in Europe (15, 19) and more recently in urine from a US dairy cow at slaughter (Hamond and Nally, unpublished data). Mare H8 was determined to be shedding *L. kirschneri*, the genome of which aligned most closely with that of the cultured isolate *L. kirschneri* serogroup Australis serovar Rushan strain H9 (Figure 1). Of note, the genomes from H8 and H9 differed by only 16 SNPs when comparing 4,565,109 shared nucleotide positions.

Serogroup Australis contains at least 14 serovars including serovars Australis and Bratislava that are commonly used as representative serovars in MAT antigen panels and both of which belong to *L. interrogans* (Supplementary Table S1). Panels of reference monoclonal antibodies typed strain H9 as serovar Rushan. The reference strain for serovar Rushan was originally isolated from a toad (*Bombina orientalis*) in China and classified as *L. noguchii* (62, 63). Average nucleotide identity (ANI) later classified it as *L. alstonii* (64). Only one serovar within the serogroup Australis is assigned to *L. kirschneri*, the reference strain for serovar Ramisi, which was originally isolated in a human patient in Kenya, *circa* 1971 (65, 66). The efficacy of bacterin vaccines in animals is dependent on the inclusion of relevant serovars associated with animal infection. Serovar status is defined using reference-agglutinating antisera or monoclonal antibodies, which target LPS, a serovar specific and protective antigen. LPS from *Leptospira* has an atypical structure from that of other gram-negative bacteria and is much less toxic likely due to modified Lipid A (67). The LPS profile of *L. kirschneri* serogroup Australis strain H9 (a strain herein associated with subclinical carriage) was compared with that of *L. interrogans* serogroup Pomona strain H44 (a strain associated with spontaneous equine abortion), *L. interrogans* serogroup Icterohemorrhagiae serovar Copenhageni strain Fiocruz_L1-130 (a strain closely related to enriched genome H2), and *L. interrogans* serogroup Australis serovar Bratislava strain PigK151 [a strain recently used in an equine challenge study (68)] to highlight differences in LPS profiles (Figure 6A). More importantly, the use of polyclonal reference antisera in immunoblots confirms antigenic differences in expression of the LPS protective antigen (Figures 6B–E) between serovars, thus demonstrating their individual status and need for inclusion in bacterins as protective antigens or in MAT panels as diagnostic antigens. The reactivity of LPS derived from *L. kirschneri* serogroup Australis strain H9 with reference antiserum specific for *L. kirschneri* serogroup Australis serovar Ramisi confirms sufficient antigenic differences with *L. interrogans* serogroup Australis serovar Bratislava, demonstrating limited cross reactivity in bacterin vaccine or MAT diagnostic antigens (Figure 6E), as confirmed by MAT titers in experimentally infected hamsters (Table 2). In contrast to LPS, proteins are highly conserved among pathogenic species and serovars (Figure 5A). The expression of known virulence factors Loa22 and LipL21 was confirmed in all strains (Figures 5B,C) (59, 69–71).

Horses are hypothesized to act as a reservoir host for serovar Bratislava in the US, according to multiple seroprevalence studies (9). However, a serovar is used in an MAT panel as a representative serovar for the serogroup so that a positive reaction is indicative of exposure to a serogroup, not specifically the serovar used in the panel (2). No direct evidence has yet been provided that serovar Bratislava persistently infects US horses (72). Recent studies have attempted to force this hypothesis by experimental infection of horses with an isolate of serovar Bratislava which was recovered from a pig a few decades ago (57), but no evidence of established infection was reported (68). In the US, serogroup Australis serovar Bratislava has only ever been isolated from the kidneys or genital tract of pigs (73, 74) and is universally reported to be a significant cause of porcine reproductive loss (1). Cows act as reservoir hosts to serogroup Sejroe serovar Hardjo, which can also colonize kidneys and/or the genital tract. Evidence of bovine exposure to serogroup Sejroe is associated with an increase in median time from calving to conception, as well as being twice as likely to fail to conceive (75). In Brazil, *Leptospira* has been detected by PCR in endometrial biopsies or vaginal fluid from mares with reproductive issues including early embryonic death and endometritis (76); thus, it would be beneficial to screen US horses for the colonization of the genital tract by *Leptospira*, whether by *L. kirschneri* serogroup Australis or other strains.

Animal leptospirosis presents with an array of clinical signs that is dependent, in part, on both the serovar of *Leptospira* and specific animal host species (77). Pathogenic mechanisms of infection are neither clearly understood nor the reason why an animal host species acts as a reservoir host for one serovar but an incidental host for others. In this study, over 90% of horses were seropositive. However, no reactivity was detected when *L. kirschneri* serogroup Australis serovar Rushan strain H9 was included in the MAT antigen panel, even though two horses (H8 and H9) are reported here as active shedders. Similarly, horse H2 had no reactive titer against serogroup Icterohemorrhagiae though it was detected in urine. The identification of seronegative reservoir hosts is not uncommon when sera are tested at 1:100, and therefore, studies with subclinical animals may test sera at starting dilutions less than 1:100 (28, 78, 79). Incidental infection is characterized by acute dissemination of *Leptospira* with limited shedding compared with reservoir hosts, which have subclinical carriage and extended shedding (80). *L. kirschneri* serogroup Australis serovar Rushan strain H9 was not detected in the liver after experimental infection in hamsters but was detected at low levels in the kidney, whereas *L. interrogans* serogroup Pomona serovar Pomona strain H44 was detected at higher levels in all the livers and kidneys (Table 2). Serovar Pomona is associated with acute disease in many animal hosts, and strain H44 is closely related to other Pomona strains isolated from multiple animals geographically distributed throughout the US (81). A total of 195 SNPs differentiate the genome of strain H44 from California sea lion isolate strain CSL4002 when comparing 4,524,948 shared nucleotide positions (Figure 2). Unique genotypes of Pomona are reported to be associated with equine abortion (11), and the advent of high-resolution genome sequencing provides opportunities to explore these associations further. Similarly, comparative genomic analyses of strains associated with acute disease compared with strains associated with subclinical carriage may provide insights into the pathogenic mechanism of infection and host adaptation.

Previous studies on equine leptospirosis have relied on EMJH media to isolate *Leptospira* from aborted and stillborn horses (8, 82). However, pathogenic *Leptospira* comprise a diverse genus of highly fastidious bacteria that require alternative media formulations since many serovars will not grow in commercial EMJH media (2, 30). Reliance on EMJH to culture leptospires from mammalian hosts is selective for only those pathogenic leptospires capable of surviving in, or adapting to, the growth media and conditions used, so alternative media formulations such as HAN are recommended. Serogroup Australis serovar Bratislava was isolated from US sows using T80/40/LH medium, not EMJH (74, 83). Historically, the isolation of pathogenic *Leptospira* from mammalian hosts has been limited to cultures incubated at 28–30°C (31). However, the use of newer media formulations such as HAN allows the isolation from mammalian hosts at 28–30°C and 37°C, a temperature that more closely emulates that encountered during host infection (30). Temperature is an important environmental signal used by *Leptospira* to regulate gene and protein expression and antigens expressed by *Leptospira* that react with equine sera (84–86). Pathogenic *Leptospira* regulate gene and protein expression during acute and chronic infection (59, 69, 87, 88), so it is hypothesized that bacterins produced at temperatures more similar to that of a host will express higher levels of cross-protective protein antigens and virulence factors (89).

Leptospirosis is a global neglected disease and paradigm of one health (90). New species and serovars continue to be identified (91, 92) highlighting its complex epidemiology and need to determine species and serovars circulating within domestic animal populations. The use of novel media formulations for *Leptospira* has facilitated the characterization of a diverse range of a new species and serovars circulating in the environment (4) and US livestock (37, 38). Similarly, we report here the identification of a new species and serovar associated with asymptomatic carriage and shedding in urine of horses, *L. kirschneri* serogroup Australis serovar Rushan. In the absence of culture, real-time PCR of urine identifies asymptomatic carriers, while DNA capture and enrichment provide comprehensive *Leptospira* genomic information. For real-time PCR, equine urine should be chilled as soon as collected and processed within 24 h for optimal sensitivity. The identification and characterization of strains associated with equine leptospirosis is essential to design efficacious diagnostic, vaccination, prevention, and treatment strategies.

## Data availability statement

The datasets presented in this study can be found in online repositories. The names of the repository/repositories and accession number(s) can be found at: https://www.ncbi.nlm.nih.gov/genbank/, BioProject ID PRJNA994138.

## Ethics statement

The animal studies were approved by the Animal Care & Use Committee at the NADC and the USDA institutional guidelines. The studies were conducted in accordance with the local legislation and institutional requirements. Written informed consent was not obtained from the owners for the participation of their animals in this study because verbal consent was provided from the animals’ agent.

## Author contributions

CH: Data curation, Formal analysis, Investigation, Methodology, Supervision, Validation, Visualization, Writing – original draft, Writing – review & editing. EA: Conceptualization, Data curation, Funding acquisition, Investigation, Methodology, Project administration, Resources, Writing – review & editing. NS: Data curation, Formal analysis, Investigation, Methodology, Software, Visualization, Writing – review & editing. KL: Data curation, Investigation, Methodology, Writing – review & editing. TA: Formal analysis, Investigation, Methodology, Writing – review & editing. EP: Data curation, Formal analysis, Investigation, Methodology, Writing – review & editing. PC: Data curation, Formal analysis, Investigation, Methodology, Writing – review & editing. JH: Data curation, Formal analysis, Investigation, Methodology, Writing – review & editing. TS: Data curation, Formal analysis, Investigation, Methodology, Writing – review & editing. HL: Data curation, Formal analysis, Investigation, Methodology, Writing – review & editing. DB: Data curation, Formal analysis, Investigation, Methodology, Writing – review & editing. JS: Data curation, Formal analysis, Investigation, Methodology, Supervision, Writing – review & editing. LS: Funding acquisition, Resources, Writing – review & editing. DW: Data curation, Formal analysis, Investigation, Methodology, Resources, Supervision, Writing – review & editing. JN: Conceptualization, Data curation, Formal analysis, Funding acquisition, Investigation, Methodology, Project administration, Resources, Supervision, Validation, Visualization, Writing – original draft, Writing – review & editing.

## Supplementary material

SUPPLEMENTARY FIGURE S1 Lower limits of detection of *Leptospira* by *lipL32* qPCR in equine urine that was processed fresh the day of collection **(A,B)**, after storage for 24 h on ice packs **(C,D)**, after storage for 48 h on ice packs **(E,F)**, or after being frozen at −20^o^C **(G,H)**. Samples **B,D,F,H** were centrifuged at 900 × g for 10 min to remove “sludge” prior to processing.

SUPPLEMENTARY FIGURE S2 Whole-genome dendrogram based upon pairwise MASH distances of *Leptospira* genomes, showing mare sequences H2, H8, H9, and H44 within the *Leptospira* P1 clade (highlighted in blue) (3). Enriched and isolate genomes for strains H8 and H9 were placed in the *L. kirschneri* clade, whereas strains H2 and H44 grouped with *L. interrogans*. The dendrogram includes genomes of 66 *Leptospira* strains representing all known species from the P1, P2, S1 and S2 clades.

SUPPLEMENTARY FIGURE S3 Serotyping of strain H9 with monoclonal antibodies (mAb) that characteristically agglutinate serovars from serogroup Australis. Titers of reactivity for each mAb are provided for *L*. *kirschneri* serogroup Australis strain H9 and reference strain *L*. *noguchii* serogroup Australis serovar Rushan strain 507. Reciprocal titers are shown on the *y*-axis; mAb number is shown on the *x*-axis.

SUPPLEMENTARY FIGURE S4 Serotyping of strain H44 with monoclonal antibodies (mAb) that characteristically agglutinate serovars from serogroup Pomona. Titers of reactivity for each mAb are provided for *L*. *interrogans* serogroup Pomona strain H44 and reference strain *L*. *interrogans* serogroup Pomona serovar Pomona strain Pomona. Reciprocal titers are shown on the *y*-axis; mAb number is shown on the *x*-axis.

SUPPLEMENTARY FIGURE S5 A maximum likelihood phylogeny of 51 *L. interrogans* reference genomes together with *Leptospira* genomic DNA from strain H2 and H44 (highlighted with blue text). The tree was inferred by IQ-TREE from a concatenated SNP alignment of 248,698 positions out of a core genome size of 2,688,796nts. *Leptospira* from strain H2 falls among other *L. interrogans* serovar Copenhageni genomes, whereas strain H44 groups among other *L. interrogans* serovar Pomona genomes. GenBank accession numbers for reference genomes are included in the annotations. The phylogeny is rooted with *L. kirschneri* strain RedPanda1.
