# Supplementary material for: Identification of equine mares as reservoir hosts for pathogenic species of Leptospira
Source: Front Vet Sci. 2024 May 9;11:1346713. doi: 10.3389/fvets.2024.1346713 (PMC11112012; doi:10.3389/fvets.2024.1346713)
Supplement: Supplementary file 2 [file Data_Sheet_1.PDF]

Supplementary Figure 1

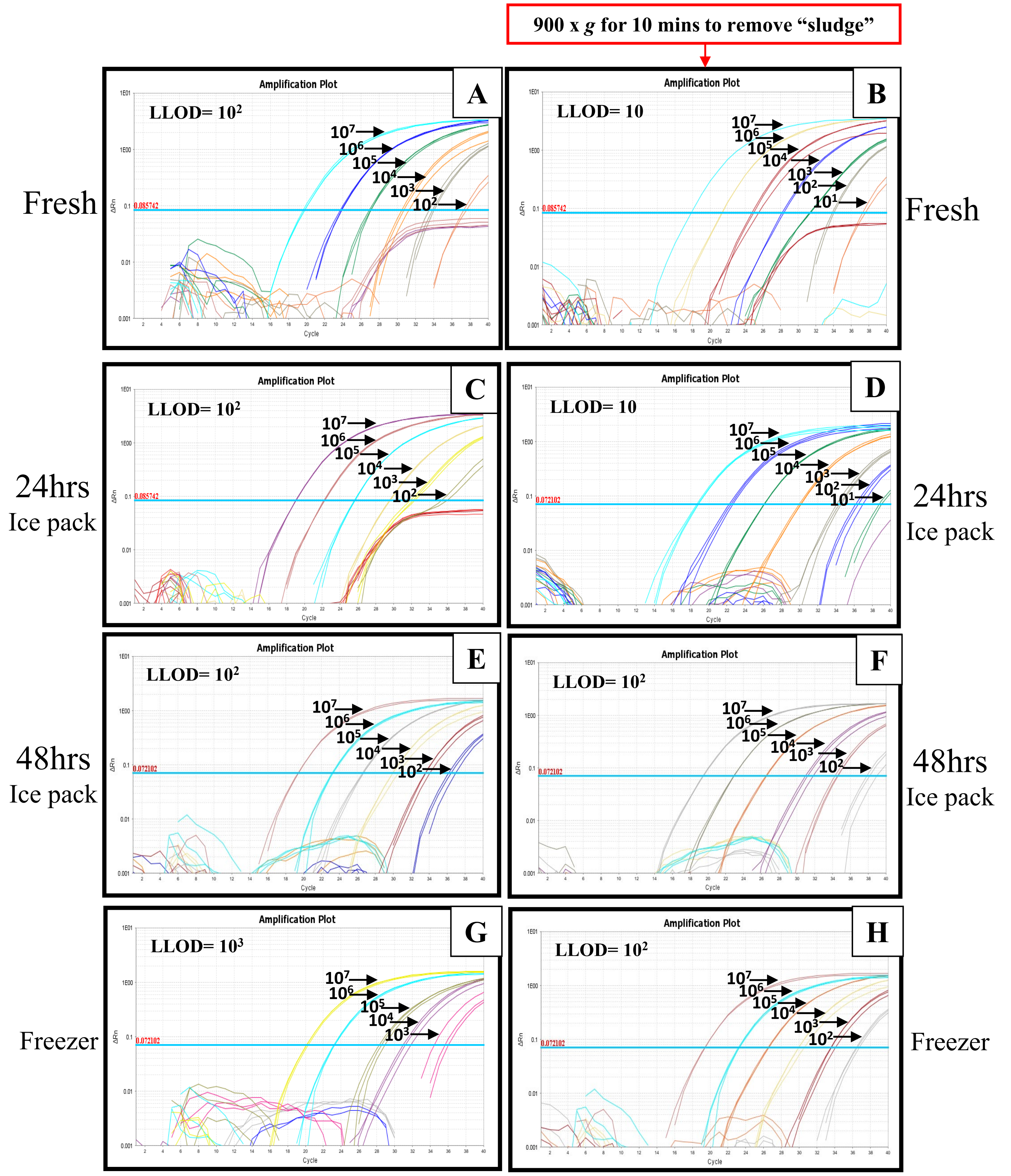

Supplementary Figure 2

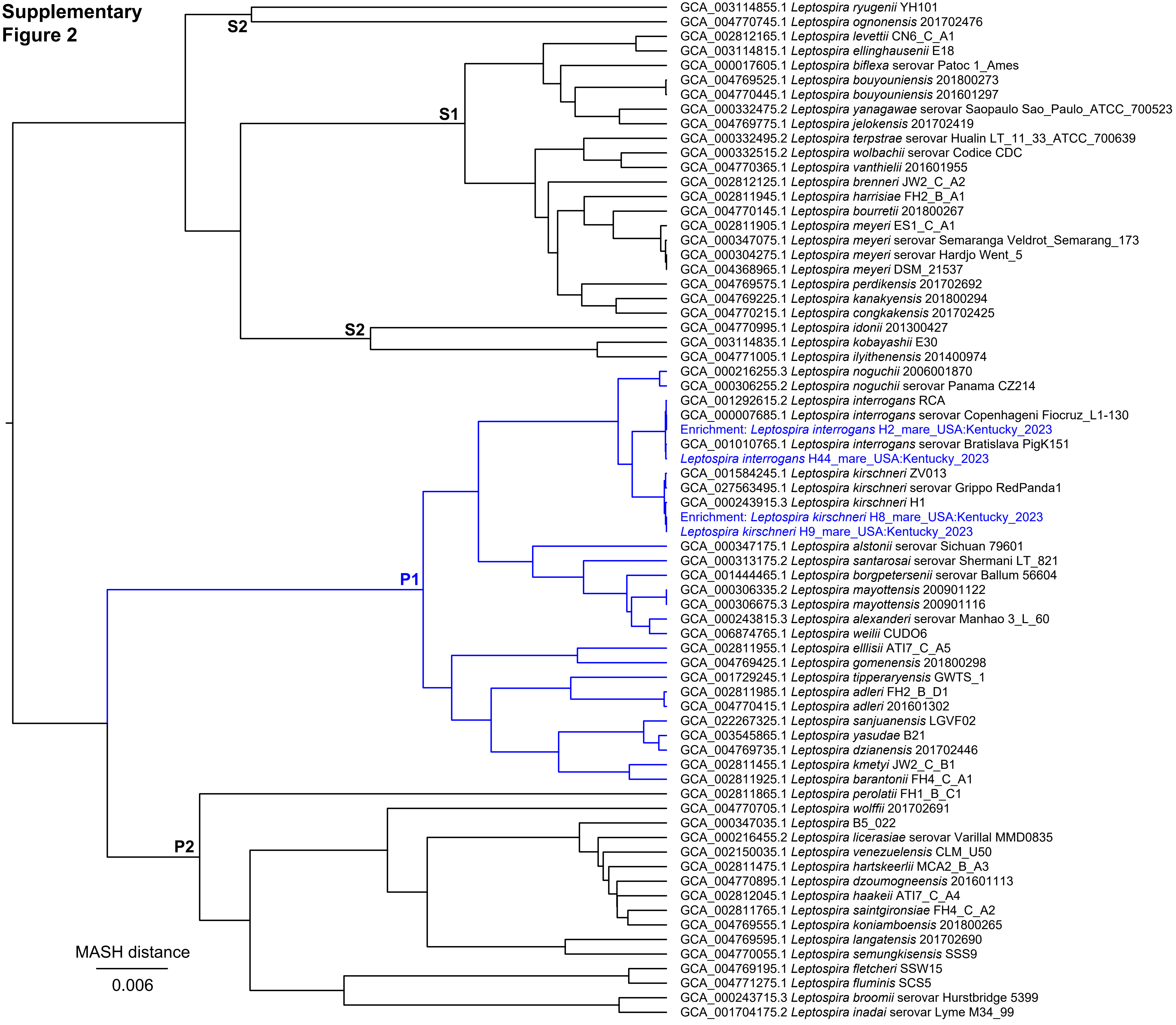

Supplementary Figure 3

Strain H9

mAb	CODE	TITRE
1	F81C3	10
2	F81C4	10
3	F81C5	10
4	F81C6	10
5	F81C8	10
6	F90C4	10
7	F90C5	10
8	F90C8	5120
9	F90C9	10
10	F132C2	10
11	F132C7	10

HISTOGRAM

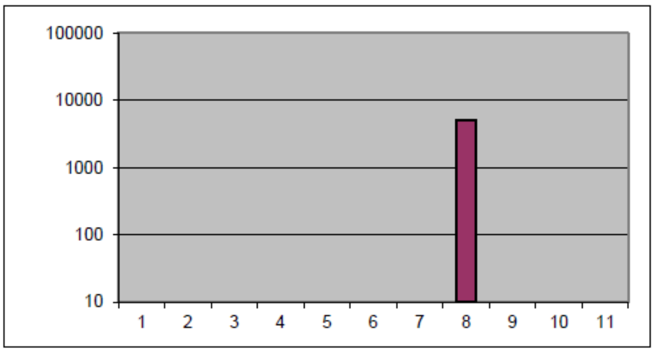

Reference strain: Serogroup Australis, Serovar Rushan, Strain 507

mAb	CODE	TITRE
1	F81C3	10
2	F81C4	10
3	F81C5	10
4	F81C6	10
5	F81C8	10
6	F90C4	10
7	F90C5	10
8	F90C8	5120
9	F90C9	10
10	F132C2	10
11	F132C7	10

HISTOGRAM

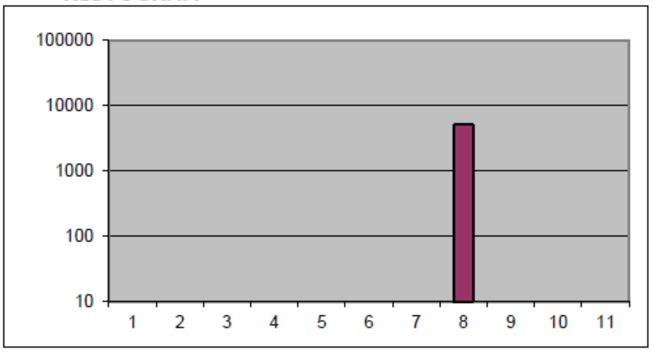

Supplementary Figure 4

Strain H44

mAb	CODE	TITRE
1	F43C9	320
2	F46C1	10
3	F46C2	10
4	F46C4	10
5	F46C5	10
6	F46C9	10
7	F46C10	10
8	F48C1	10
9	F48C3	10
10	F48C6	10240
11	F58C1	10
12	F58C2	10
13	F61C7	320

HISTOGRAM

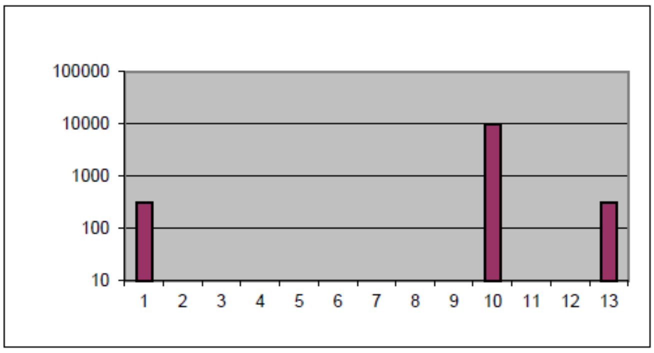

Reference strain: Serogroup Pomona, Serovar Pomona, Strain Pomona

mAb	CODE	TITRE
1	F43C9	160
2	F46C1	10
3	F46C2	10
4	F46C4	10
5	F46C5	10
6	F46C9	10
7	F46C10	10
8	F48C1	10
9	F48C3	10
10	F48C6	1280
11	F58C1	10
12	F58C2	10
13	F61C7	80

HISTOGRAM

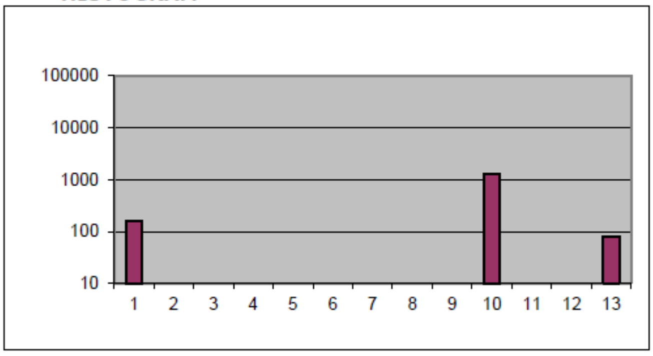

Supplementary Figure 5

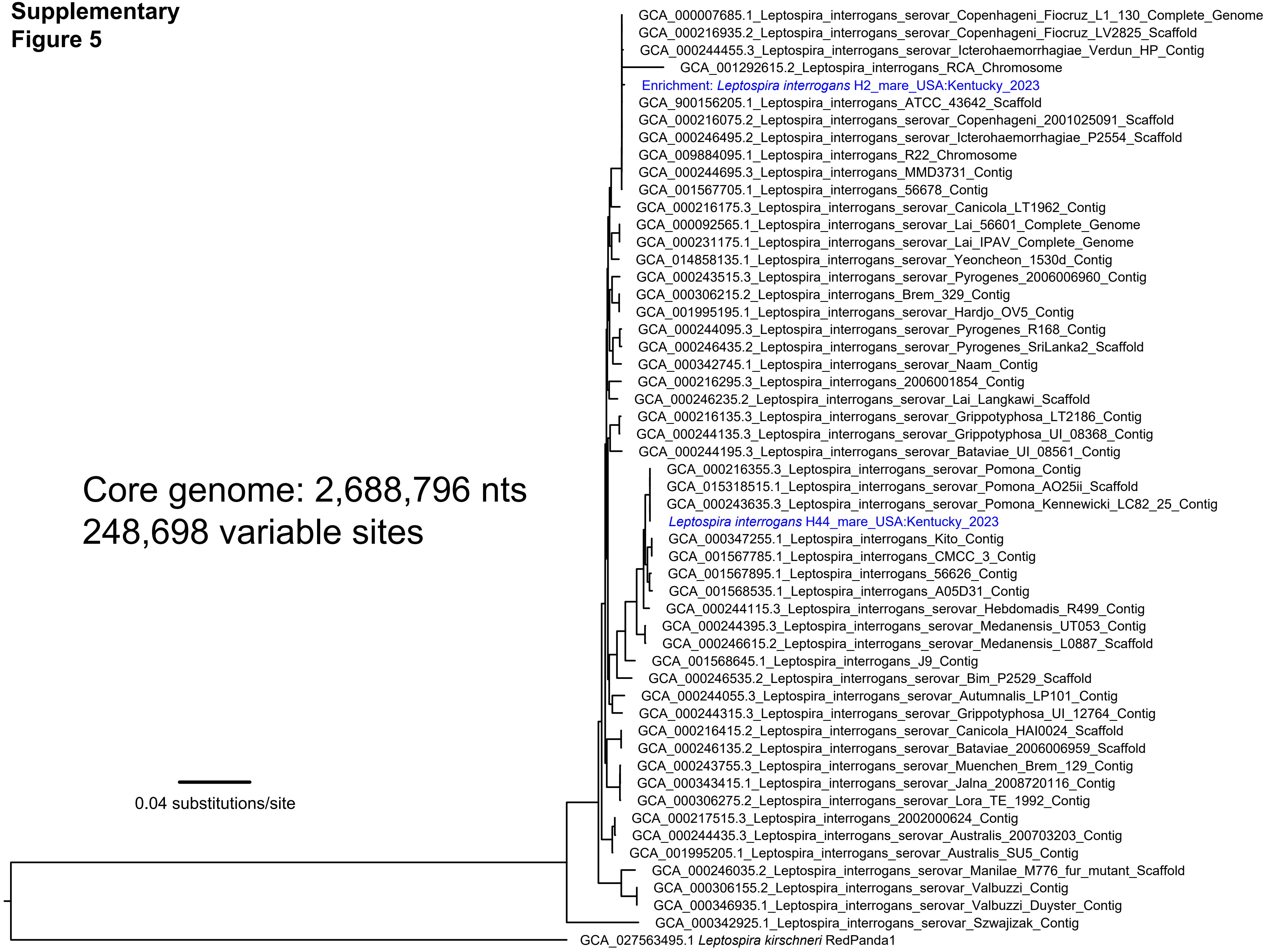

Supplementary Table 1: Panel of antigens used in the microscopic agglutination test (MAT).

Species	Serogroup	Serovar	Strain
L. interrogans	Australis	Bratislava	Jez Bratislava
L. interrogans	Australis	Australis	Ballico
L. interrogans	Autumnalis	Autumnalis	Akiyami A
L. borgpetersenii	Ballum	Ballum	S 102
L. interrogans	Bataviae	Bataviae	Van Tienen
L. interrogans	Canicola	Canicola	H. Utrecht IV
L. kirschneri	Cynopteri	Cynopteri	3522C
L. interrogans	Djasiman	Djasiman	Djasiman
L. interrogans	Grippotyphosa	Grippotyphosa	Andaman
L. interrogans	Hebdomadis	Hebdomadis	Hebdomadis
L. interrogans	Icterohaemorrhagiae	Copenhageni	M 20
L. interrogans	Mini	Szwajizak	Szwajizak
L. interrogans	Pomona	Pomona	Pomona
L. interrogans	Pyrogenes	Pyrogenes	Salinem
L. interrogans	Sejroe	Hardjo	Hardjoprajitno
L. borgpetersenii	Sejröe	Sejröe	M 84
L. borgpetersenii	Tarassovi	Tarassovi	Perepelitsin
L. tipperaryensis	ND	Room 22	GWTS#1

ND: Not determined

Supplementary Table 2: Panel of reference antisera used to identify the serogroup of equine isolates.

**Species**	**Serogroup**	**Serovar**	**Strain**
L. interrogans	Australis	Bratislava	Jez Bratislava
L. interrogans	Australis	Australis	Ballico
L. interrogans	Autumnalis	Autumnalis	Akiyami A
L. borgpetersenii	Ballum	Ballum	S 102
L. interrogans	Bataviae	Bataviae	Van Tienen
L. interrogans	Canicola	Canicola	H. Utrecht IV
L. interrogans	Grippotyphosa	Grippotyphosa	Andaman
L. interrogans	Hebdomadis	Hebdomadis	Hebdomadis
L. interrogans	Icterohaemorrhagiae	Copenhageni	M 20
L. interrogans	Mini	Szwajizak	Szwajizak
L. interrogans	Pomona	Pomona	Pomona
L. interrogans	Pyrogenes	Pyrogenes	Salinem
L. interrogans	Sejroe	Hardjo	Hardjoprajitno
L. borgpetersenii	Sejröe	Sejröe	M 84
L. borgpetersenii	Tarassovi	Tarassovi	Perepelitsin
